# Comparison of a Novel Tension Band and Patellotibial Tubercle Cerclage in the Treatment of Comminuted Fractures of Inferior Pole of the Patella

**DOI:** 10.1111/os.12616

**Published:** 2020-01-20

**Authors:** Zhi‐shan Zhang, Peng‐fei Li, Fang Zhou, Yun Tian, Hong‐quan Ji, Yan Guo, Yang Lv, Zhong‐wei Yang, Guo‐jin Hou

**Affiliations:** ^1^ Department of Orthopaedics Peking University Third Hospital Beijing China

**Keywords:** Cannulated screw, Inferior pole fracture of the patella, Patellotibial tubercle cerclage, Tension band

## Abstract

**Objective:**

To assess the therapeutic effect of a novel tension band using 3.0 mm cannulated screw combined with a titanium cable and specific shims comparatively with patellotibial tubercle cerclage in comminuted fractures of the inferior pole of the patella.

**Methods:**

The retrospective study from March 2012 to July 2017 was conducted in Peking University Third Hospital and comprised 63 patients with comminuted fractures of the inferior pole of the patella: 41 treated with new tension band using 3.0 mm cannulated screw combined with a titanium cable and specific shims (new tension band group) and 22 with patellotibial tubercle cerclage (tubercle cerclage group). Gender, age, AO/OTA fracture type, injury mechanism, inter‐fragmentary gap, and follow‐up time of patients were recorded. Two groups were compared regarding: operation time, blood loss, partial weight‐bearing time, fracture‐healed time, Bostman score and knee mobility at 12‐month follow‐up, and postoperative complications. Continuous and categorical parameters were analyzed by Mann‐Whitney *U* test and the chi‐squared test, respectively. Fisher's exact test was used for small data subsets.

**Results:**

Between the two groups, no statistically significant difference was found in mean age, gender, AO/OTA fracture type, injury mechanism, mean inter‐fragmentary gap, or mean follow‐up time (*P* > 0.05). The mean operation time of new tension band group was significantly longer than that of tubercle cerclage group (76.4 min *vs* 64.2 min, *P* = 0.006), while there was no significant difference in blood loss. After surgery, new tension band group had a significantly earlier mean partial weight‐bearing time (5.2 weeks *vs* 7.4 weeks, *P* < 0.001) and fracture‐healed time (9.6 weeks *vs* 11.6 weeks, *P* < 0.001). At 12‐month follow‐up, patients of new tension band group had a significantly higher mean Bostman score (28.5 *vs* 25.8, *P* < 0.001) and knee mobility (126.7 *vs* 117.3, *P* < 0.001). Ten complications related with internal fixation were found in tubercle cerclage group including two cases of loose internal fixation, two cases of cerclage breakage, and six cases of low patella position who undertook secondary operation. No complications were found in new tension band group (0 in 41 *vs* 10 in 22, *P* < 0.001).

**Conclusion:**

Patients with comminuted fractures of the inferior pole of the patella treated with a novel tension band experienced a longer operation time, but earlier partial‐weight‐bearing and fracture‐healed time, better clinical outcomes at 12‐month follow‐up, and less complications. It should be considered an alternative therapy for the treatment of distal pole patellar fractures.

## Introduction

The patella is the biggest sesamoid bone in the human body, situated within the tendon of the quadriceps femoris muscle; it is functionally part of the knee extension system. Patella fractures result in a functional disability of the knee extension system, and constitute approximately 1% of all fractures^1^.

The inferior pole is an anterior cortical extension of the patella body without articular cartilage. It is the origin of the patellar tendon and bears a high stress. Inferior pole fractures of the patella account for 9.3%–22.4% of all patellar fractures^2^, and are usually completely extra‐articular, comminuted, and usually measuring less than 15 mm in vertical length. It is postulated that inferior pole avulsion fractures are more likely to occur when the knee is flexed near 90°, and the patella is dynamically locked in the femoral condylar groove^3^. Inferior pole fractures are usually treated surgically and the goal is to achieve quadriceps extensor mechanism restoration rather than anatomical articular reduction.

Complete patellectomy for comminuted fractures of patella was no longer recommended as studies have demonstrated a high rate of subjective dissatisfaction and poor knee function in patients^4^. But considering the particularity of the fractures of the inferior pole, excision of the inferior patella and reattachment of patella tendon may be performed due to the difficulty of fixation and maintenance of reduction. Saltzman *et al*.^5^ reported 40 patients who had been followed for an average of 8.4 years after partial patellectomy as a treatment for displaced patellar fractures; the overall result was rated as excellent in 20 patients, good in 11, fair in six, and poor in three. There was a significant statistical correlation between the type of fracture and the outcome. Kastelec *et al*.^6^ reported 14 patients with inferior pole fractures of the patella treated with pole resection and tendon reattachment, obtaining good clinical outcome at last follow‐up, but requiring postoperative immobilization in a cast for an average of 6.5 weeks. Furthermore, the change of length of the patellar tendon after pole resection might disrupt the normal physiology of the patellofemoral joint and cause long‐term problems^5^, ^7^. In a retrospective study containing 52 patients, though functional outcomes seemed to be similar to open reduction and internal fixation (ORIF), partial patellectomy with trans‐osseous pull‐out sutures and augmentation with cerclage wire required immobilization, might result in anterior knee pain, abnormal patella height and loss of range of motion^8^. More importantly, outcomes of the above articles should be interpreted with caution because they were all small‐sample studies that didn't consider the diversity and progress of internal fixations. The concept that the pole fragments should be preserved as much as possible in treatment of inferior pole fracture is getting its popularity among orthopaedic surgeons, and excision of the inferior patella is considered as a secondary option or a revision surgery for implant failures.

Recently, most surgeons attempted to retain some of the fragments of inferior pole and many treatment methods for retaining inferior pole fragments were reported, mainly in various forms of internal fixation, such as wire looping, basket plate, and anchor suture fixation^2^, ^9^, ^10^, ^11^. Patellotibial tubercle cerclage with decreasing tension band method were also introduced as an effective method to treat the comminuted inferior polar fracture of patella with few complications and favorable recovery^12^. The tension band method has been widely used in treating patellar fractures^13^, and the technique using this tension band method targeted for inferior polar fracture was reported^14^. In our institute, we also preferred the tension band method and developed a novel tension band using 3.0 mm cannulated screw combined with a titanium cable and specific shims for comminuted fractures of the inferior pole of the patella. However, every technique had its shortcomings, and no one technique was universally used; although there were some researchers comparing the outcomes of their internal fixation in ORIF to partial patellectomy^6^, ^11^, few comparisons of clinical outcomes of different internal fixations were reported.

Therefore, this retrospective study assessed the clinical outcome of a novel tension band using 3.0 mm cannulated screw combined with a titanium cable and specific shims comparatively with patellotibial tubercle cerclage in comminuted fractures of the inferior pole of the patella.

The aim of the present study was to provide orthopaedic surgeons with: (i) an overview of different ORIF techniques for treating fractures of the inferior pole of the patella and their advantages and disadvantages; (ii) an introduction to the surgical technique of the novel tension band as an effective therapy for comminuted fractures of the inferior pole of the patella; and (iii) a summary of the difficulties of this surgical technique and proposed solutions.

Patients treated with novel tension band were defined as new tension band group, and those who were treated with patellotibial tubercle cerclage were defined as tubercle cerclage group. We compared two groups regarding operation time, blood loss, partial weight‐bearing, fracture‐healed time, Bostman score and knee mobility at 12‐month follow‐up, and postoperative complications.

## Patients and Methods

### *Patients Data*

This retrospective study included patients at our institution between March 2012 and July 2017. Inclusion criteria were: (i) patients experienced a patella fracture within 3 days; (ii) physical examination indicated patients with functional disability of the knee extension system due to patella fracture; (iii) X‐ray indicated that fractures were inferior pole patellar, including AO/OTA 34‐A1 fracture (inferior pole avulsion fracture with intact patellar articular surface) and AO/OTA 34‐C1.3 fracture (inferior pole patellar fracture primarily with a transverse fracture line, involving little articular surface)^15^; (iv) displaced fractures needed surgical treatment; and (v) received ORIF with novel tension band or patellotibial tubercle cerclage.

Exclusion criteria were: (i) any difficulties to ambulate or functional limitations before injury; (ii) pathological fracture, delayed fracture, stress fracture, or periprosthetic fracture; and (iii) follow‐up less than 12 months.

The study was approved by the Ethics Committee of the hospital and was subject to its supervision. Informed consent was obtained from all patients or their family members, and the study conformed to the provisions of the Declaration of Helsinki (as revised in Brazil in 2013).

Patient demographics and clinical characteristics were recorded, including: gender, age at the time of operation, the AO/OTA classification, and injury mechanism. The preoperative inter‐fragmentary gap was also recorded, defined as the distance from the upper edge of the inferior fragment to the lower edge of the superior fragment of the patella on lateral position X‐ray of knee.

### *Surgical Techniques*

Patients were divided into the new tension band group and patellotibial tubercle cerclage group, and undertook different surgical procedures. Operation was considered when articular displacement was greater than 2 mm or fragment separation exceeded 3 mm as assessed by radiography.

All patients were treated with spinal anesthesia. Patients were placed in the supine position on a radiolucent table, and a pneumatic tourniquet was used in all cases. We performed a standard midline longitudinal incision extending from the upper end of the patella to patellar tendon. Full thickness skin flaps were raised medially and laterally to expose the transversely ruptured retinaculum, fracture, and patellar tendon. Fracture fragments were identified and irrigated with saline to remove hematomas. In inferior pole fractures of the patella, comminution was common, and care was taken to preserve soft tissue attachment of patellar tendon to distal fragments.

**Figure 1:**
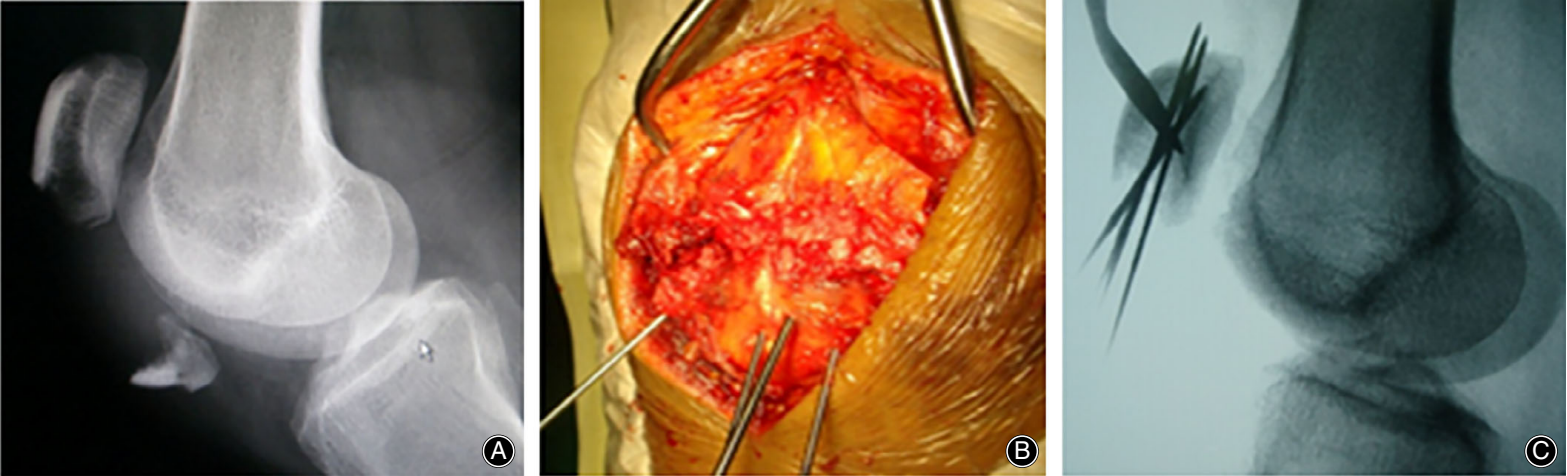
(A) Male, 40 years old, slip accident, comminuted inferior pole fractures of the right patella (AO/OTA type: A1). (B) Fracture reduction, multiple K‐wires (φ1.5 mm and φ1.2 mm) temporary fixed. (C) Intraoperative fluoroscopy showing satisfactory fracture reduction.

**Figure 2:**
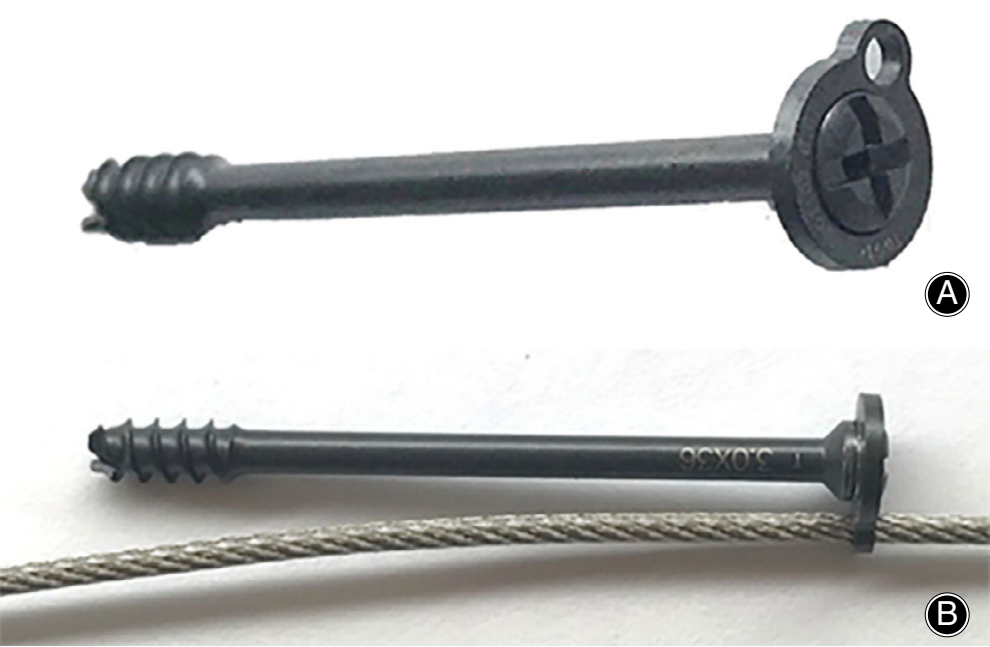
(A) Aφ3.0 mm cannulated screw with a specially designed shim. (B) A φ1.3 mm titanium cable passed through the small hole of the shim; the cannulated screw, shim and titanium cable constitute a stable, fixed construction.

**Figure 3:**
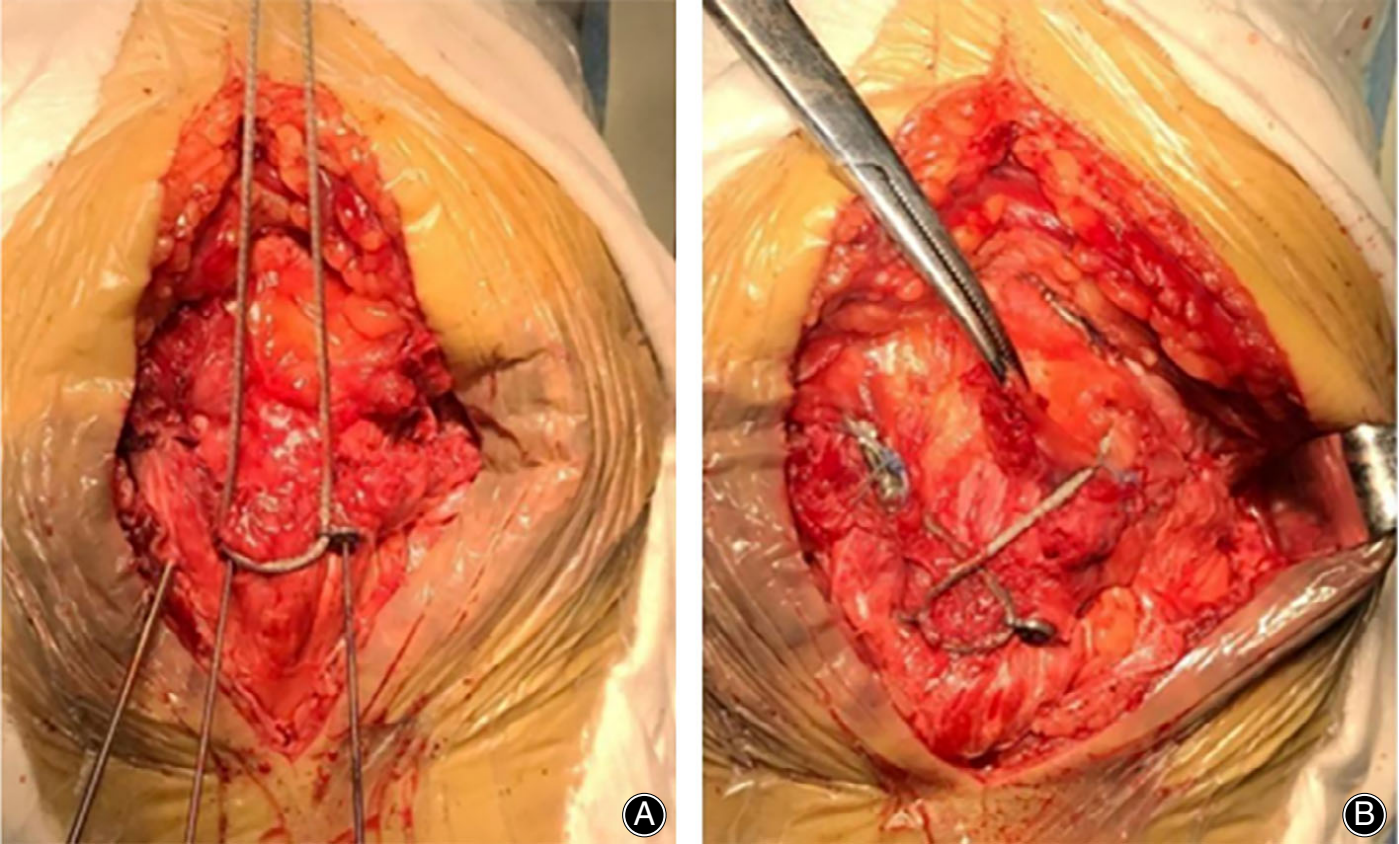
(A) Insertion of the φ3.0 mm cannulated screw with the special shim along the guide pin. The titanium cable passes through the small hole of the shim, then tightens the titanium cable and the cannulated screw. (B) The titanium cable passes through the bone hole of the widest part of the proximal patella, then tightens the titanium cable to form the figure‐of‐eight tension band at the anterior cortex of the patella, fixing the titanium cable with cable clamps.

**Figure 4:**
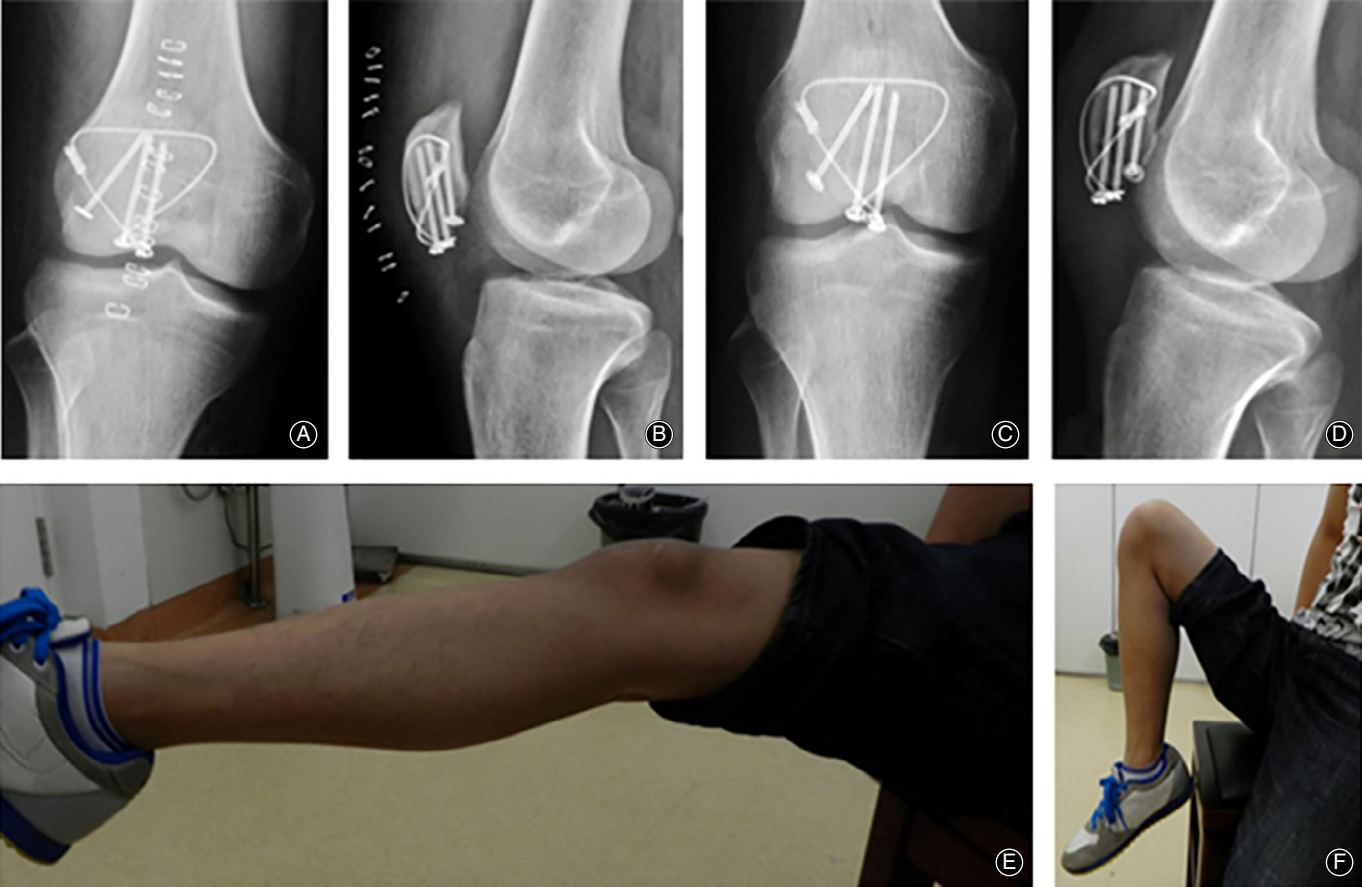
(A) and (B) Anteroposterior and lateral radiographs of the knee postoperatively. (C–F) A total of 2 months postoperatively, the fracture was well healed; the right knee joint functioned normally, and normal walking function was restored (Bostman score: 30 points).

In the novel tension band group, with the knee in extension, the fragments were clamped to reduce the fracture with small towel clamps. We drilled two K‐wires (1.5mm diameter) in parallel from the inferior pole to the superior pole of the patella. The K‐wire was inserted as far as possible from the center of the fracture fragment, 30° from the coronal plane of the patella to avoid the articular surface. The K‐wires did not penetrate the cortex of the superior patellar pole (Fig. 1). The position of the K‐wire was determined by intraoperative fluoroscopy. One K‐wire was first removed, and a cannulated screw guide pin was inserted along the pin tract. After measuring its depth, a 3.0‐mm titanium cannulated compression screw was placed with a special shim (Weigao Inc. China) along the guide wire. The shim was specially designed, and the titanium cable could pass through its small hole (Fig. 2). We then inserted a second cannulated screw with the special shim, and alternately tightened the screws. The screw was inserted into the proximal fracture fragment, without exposing its head from the proximal end of the patella. Next, the guide wire was removed, and a 1.3‐mm‐diameter titanium cable (consisting of a total of nine strands; Zimmer Inc., Warsaw, IN, USA) was threaded through the small hole of the special shim. The widest part of the proximal patella was drilled transversely, and the titanium cable went through the bone hole (Fig. 3). We then tightened the titanium cable to form the figure‐of‐eight tension band at the anterior cortex of the patella, fixed the titanium cable with cable clamps, and cut off the excessive titanium cable (Fig. 4). Stability of reconstructed patella was tested by placing the knee through a range of motion; a layered wound closure without suction drain was performed.

**Figure 5:**
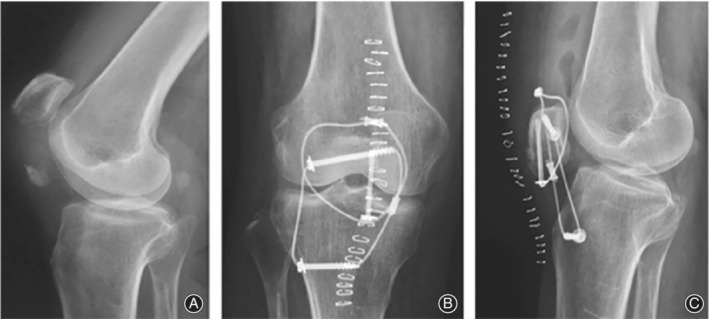
(A) Female, 55 years old, slip accident, right comminuted inferior pole fracture of the patella (AO/OTA type: A1). (B, C), Fracture reduction and internal fixation, 3.0 mm cannulatedscrew to fix the inferior pole fracture fragment. Patellotibial tubercle cerclage was performed. Postoperative X‐ray showing low position of the patella.

In the patellotibial tubercle cerclage group, we used a similar method to reduce the inferior patellar fracture. Precisely, 3.0‐mm titanium cannulated or anchor screws or sutures were used to fix the small fragments. The extensor tendon and retinaculum were repaired with #1 Vicryl. The titanium cable circumscribed the patella. The proximal end of the patella was drilled transversely at the widest point. Another bone hole was drilled at the base of tibial tuberosity under direct view and as vertical to the long axis of the tibia as possible. The titanium cable was passed through the two bone holes, and the knee joint was flexed at a 30° position to form a tension band. A layered wound closure without suction drain was performed (Fig. 5).

All surgeries were operated by Zhi‐shan Zhang and Fang Zhou.

### *Postoperative Procedure*

No external splints were used in the novel tension band group. The patients performed quadriceps femoris contraction exercises soon after the operation, passive joint flexion and extension exercises 2 days postoperatively, and active joint flexion and extension exercises 7 days after surgery. Weight bearing was allowed. Knee flexion was allowed less than 45° in the first week and increased gradually to 90° in the second week. Patients were encouraged to gain full range of motion within 4 weeks.

**Figure 6:**
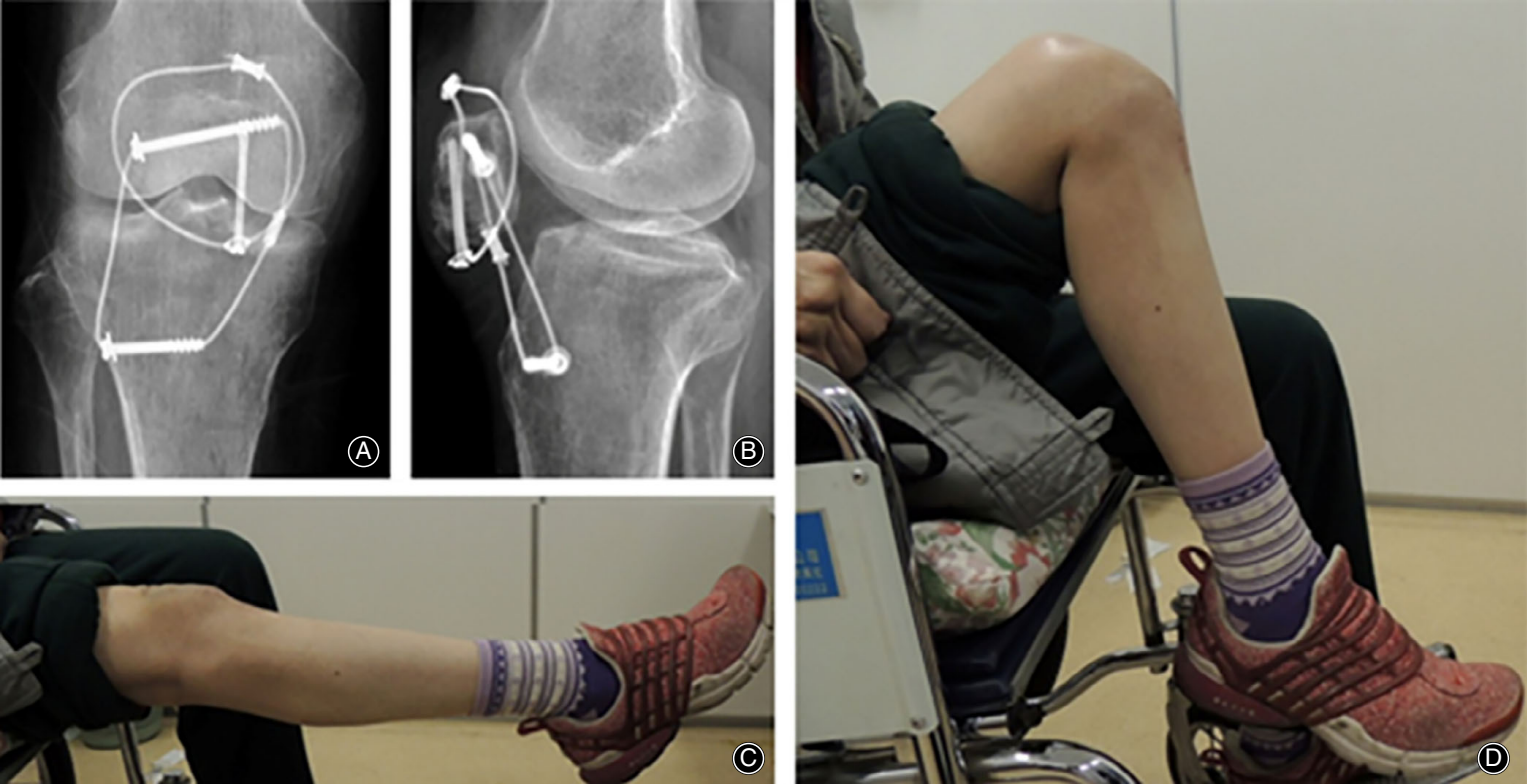
(A) and (B) A total of 8 weeks postoperatively, X‐ray radiography showed good internal fixation and fracture healing. (C, D) Knee activity was 10° to 100° 8 weeks postoperatively, without weight‐bearing walking.

The patients of the patellotibial tubercle cerclage group need fixation splints for 3 weeks after the operation. The patients performed quadriceps femoris contraction exercises soon after the operation. Three weeks later, they were allowed passive and active joint exercises. After 2 weeks, partial weight‐bearing was allowed. Patients were encouraged to gain full range of motion within 8 weeks after operation (Fig. 6).

### *Clinical Assessment*

Operation time was recorded and defined as time from skin cut to sewed. Blood loss during surgical procedure was also recorded. Patients were reexamined at 1, 2, 3, 6, and 12 months postoperatively, including clinical examinations and radiographs.

#### *Partial Weight‐Bearing and Fracture‐Healed*

Partial weight‐bearing was defined as unpainful walking with aid of an auxiliary tool, usually a single cane. From 2 weeks after surgery, patients of the novel tension band group were recommended to step on a weighing scale with the injured leg every day while being supported by someone (by the patient's arm) and record the maximum weight without causing pain. Patients of the patellotibial tubercle cerclage group were asked for the same thing since fixation splints were removed. When maximum weight came up to 20 kg, partial weight‐bearing was allowed. Fracture‐healed was defined as absence of local pain or tenderness, ability to walk well without help, and evidence of trabecular bone growing across the fracture line.

#### *Bostman Score*

The Bostman score was used to assess functional recovery after surgery for fracture of the patella^3^, including knee range of motion (six points), pain (six points), working condition (four points), quadriceps muscle atrophy (four points), auxiliary tools (four points), joint effusion (two points), soft legs (two points), and stairs (two points). The total score is 30 points: 28–30 points, excellent; 20 to 27 points, good; and less than 20 points, poor.

#### *Complications*

Complications related to the implant included loosening of internal fixation, deep infection, revision operation due to breakage of the reduction band, and revision operation due to low patella position causing unbearable pain when the knee flexed. When breakage or unbearable pain caused by low patella position was found in patients, they were commended a lower‐limb braking on extension position. After fracture healed in these patients, a secondary operation would be performed to remove implant.

For each patient, the time from operation to partial weight‐bearing and fracture‐healed were recorded, respectively. Knee range of motion was assessed, the Bostman score was evaluated at each follow‐up, and the results at 12‐month follow‐up were considered the final functional outcomes.

#### *Statistical Analysis*

SPSS version 23.0 (Chicago, IL, USA) was used to compare the demographic data and clinical outcomes between the groups. Continuous and categorical parameters were analyzed by Mann‐Whitney *U* test and the chi‐squared test respectively. Fisher's exact test was used for small data subsets (*n* < 5). *P* < 0.05 was considered statistically significant.

## Results

### *Patients Data*

Overall, 63 fractures reviewed met the inclusion criteria for the study: 41 patients in the novel tension band group and 22 patients in the patellotibial tubercle cerclage group (Table 1). No statistically significant difference was found in gender (22 female to 19 male *vs* 14 female to 8 male; *P* > 0.05), mean age (56.7 years *vs* 57.4 years; *P* > 0.05), AO/OTA fracture type (24 A1‐to‐17 C1.3 *vs* 17 A1‐to‐5 C1.3; *P* > 0.05), injury mechanism (40 fall to 1 others *vs* 21 fall to 1 others; *P* > 0.05), mean inter‐fragmentary gap (16.8 mm *vs* 15.8 mm; *P* > 0.05), or mean follow‐up time (18 months *vs* 15 months; *P* > 0.05).

**Table 1:** Patients data

Indexes	Patellotibial tubercle cerclage (22 cases)	New tension band (41 cases)	*P* value
Gender [cases (%)]			0.594
Female	14 (63.6)	22(53.6)	
Male	8(36.3)	19 (46.3)	
Age (years, mean ± SD)	57.4 ± 13.7	56.7 ± 13.8	0.847
AO/OTA Type			0.172
A1	17 (77.3)	24 (58.5)	
C1.3	5 (22.7)	17 (41.5)	
Injury Mechanism			1.000
Simple Fall	21 (95.5)	40 (97.6)	
Others	1 (4.5)^a^	1 (2.4)^b^	
Inter‐fragmentary Gap (mm, mean ± SD)	15.8 ± 8.0	16.8 ± 5.4	0.495
Follow‐up time(months, mean ± SD)	15.0 ± 5.2	18.0 ± 11.6	0.370

a A traffic injury.

b A high falling injury.

### *Clinical Outcomes*

The mean operation time of new tension band group was significantly longer than that of tubercle cerclage group (76.4 min *vs* 64.2 min, *P* = 0.006); there was a longer time of 12.2 min. While there was no significant difference in blood loss (12.5 mL *vs* 13.2 mL, *P* = 0.006).

#### *Partial Weight‐Bearing and Fracture‐Healed*

After surgery, new tension band group had a significantly earlier mean partial weight‐bearing time (5.2 weeks *vs* 7.4 weeks, *P* < 0.001) and fracture‐healed time (9.6 weeks *vs* 11.6 weeks, *P* < 0.001) (Table 2). New tension band group's weight‐bearing time was shorter 2.2 weeks than that of tubercle cerclage group. New tension band group's fracture‐healed time was shorter by 2 weeks than that of tubercle cerclage group.

**Table 2:** Clinical outcomes (mean ± SD)

Indexes	Patellotibial tubercle cerclage (22 cases)	New tension band (41 cases)	*P* value
Operation time (min)	64.2 ± 10.8	76.4 ± 18.0	0.006
Blood loss (mL)	12.5 ± 3.3	13.2 ± 3. 5	0.465
Partial weight‐bearing^a^(weeks)	7.4 ± 0.9	5.2 ± 1.3	<0.001
Fracture‐Healed^b^(weeks)	11.4 ± 1.3	9.6 ± 1.9	<0.001
Bostman Score	25.8 ± 1.3	28.5 ± 1.6	<0.001
Knee mobility (°)	117.3 ± 6.9	126.7 ± 6.6	<0.001

a Time from operation to partial weight‐bearing.

b Time from operation to fracture‐healed.

#### *Bostman Score*

At 12‐month follow‐up, patients of new tension band group had a significantly higher mean Bostman score (28.5 *vs* 25.8, *P* < 0.001) and knee mobility (126.7° *vs* 117.3°, *P* < 0.001) (Table 2), and there was a higher value of 2.7 and 9.4° in new tension band group.

#### *Complications*

**Figure 7:**
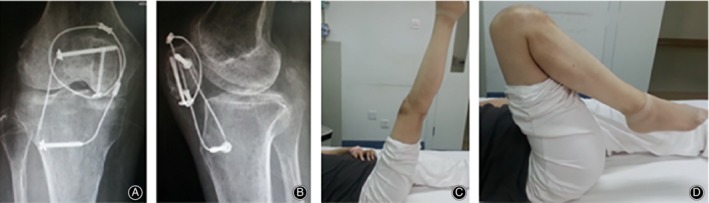
(A) and (B) At postoperative 5 months, patellotibial tubercle cerclage breakage. (C, D) The patient's knee activity was 0°–125°, walking with soft leg symptoms, knee pain when climbing stairs; daily work intensity was reduced, with a Bostman score of 26 points; 5 months after surgery, a second surgery was performed to remove the implants.

No deep infection was found in 63 patients. Ten complications related with internal fixation in 22 patients were found in tubercle cerclage group, including two cases of loose internal fixation, two cases of cerclage breakage, and six cases of low patella position who undertook secondary operation (Fig. 7). No complication was found in new tension band group (0 in 41 *vs* 10 in 22, *P* < 0.001).

## Discussion

Inferior pole fractures of the patella account for 9.3%–22.4% of all patellar fractures^2^, and various surgical procedures to preserve fragments of the inferior pole is gaining popularity recently^8^, ^9^, ^10^, ^14^. However, it remains doubtful which surgical technique is superior.

### *Previous Techniques*

Conventional wire fixation with K‐wires or screws is suitable for big fragments. Problems with this fixation technique include loss of reduction and implant migration caused by soft tissue atrophy and lack of fixation rigidity. Prominent Kirschner pins and twisted wire ends in this subcutaneous location often cause soft tissue irritation^16^, ^17^. Inferior pole patellar fracture fragments are small and comminuted. The fracture line is relatively far from the upper edge of the patella. It is difficult to place the K‐wire at the ideal position of the lower part of the patella; the fixation strength is not enough, so it takes a long time to fix the injured knee postoperatively. In addition, the end of the K‐wire is too short for easy wire removal, and the fracture is displaced. The needle tail is too long to stimulate the local tissue; this leads to local discomfort and formation of burs, affecting postoperative joint function and causing other adverse effects.

The tension band with the cannulated screw is the same as the K‐wire tension band^18^, but for comminuted small inferior pole fractures of the patella, φ4.0 mm cannulated screws are difficult to achieve ideal positioning in the inferior pole of the patella. While inserting the φ4.0 mm cannulated screw, inferior pole fractures will be further aggravated so that a sufficient fixation effect cannot be achieved. Chang *et al*.^14^ reported an average 17° loss of knee flexion in 50% of patients after open reduction and internal fixation of displaced inferior pole patella fractures using anterior tension band wiring through the cannulated screws. As this method does not cover the middle of the patellar apex, the most plausible explanation for these results is insufficient fixation of the comminuted distal pole.

Basket plate osteosynthesis has reported for the treatment of severe comminuted fractures of the inferior pole of the patella^2^, ^19^, ^20^. The basket plate can collect the fragments of the distal pole of the patella together into the basket‐shaped plate, and is fixed to the proximal patella by four or six cancellous screws. This can provide stable osteosynthesis with normal height of the patella, immediate mobilization, and early weight‐bearing ability. However, this specific plate is not available in all institutions, and injury to the patellar tendon and the relative bulk of the plate with the thin soft tissue layer over the anterior patella are the main drawbacks. Krkovic *et al*.^21^ conducted a biomechanical study, and found that the basket plate could cause significant shortening and rupture of the patellar tendon.

A separate vertical wiring technique for comminuted fractures of the distal patella introduced by Yang *et al*.^10^ showed higher fixation strength compared with tension‐band wiring in a biomechanical study. However, the operation was complicated. The knee was immobilized in a long leg splint for 2 days after the operation and protected with a brace for a period of 1 month. Postoperative follow‐up also revealed displacement of the lower pole fracture. Moreover, concerns about the holding power of separate vertical wires for comminuted small fragments remained, especially in the elderly. The force loaded to the completely extended quadriceps muscle is 316 N, and vertical wiring provides failure load at 216 N. Song *et al*.^22^ reported data for mechanical testing using a modified technique (augmented vertical wiring with cerclage wire), with a higher average ultimate failure load of 325 N. The combined procedure showed better fixation results compared with separate vertical wiring alone; however, they experienced four cases of cerclage wire breakage out of 21 patients. Oh *et al*.^23^ reported modifying a separate vertical wiring technique by adding Krackow suture for patellar tendon to improve immediate fixation stability.

### *Novel Tension Band*

The new tension band using the φ3.0 mm cannulated screws with a special shim and the cable overcame the shortcomings of the above methods. This method has the following advantages: (i) it preserves the integrity of the patella and restores the original anatomical relationship of the knee extension device; (ii) after reducing the inferior pole fracture of the patella, the φ3.0 mm cannulated screw is inserted from the distal to proximal end; the screw does not need to penetrate the proximal bone, which reduces the difficulty of insertion from the proximal to distal end of the patella; the surgical procedure is simple; (iii) the φ3.0 mm cannulated screw can fix the distal pole fracture fragment reliably, avoiding that a larger diameter of the screw further crushes the inferior pole fracture block; the new special shim increases the area of the screw cap; (iv) the new tension band using the φ3.0 mm cannulated screw with the special shim and the cable can fix the inferior pole fracture of the patella reliably; therefore, knee function exercises can begin early; (v) the new tension band system has little effects on the patellar tendon, and all materials are titanium based; secondary removal operation is not required; (vi) the titanium cable is more flexible than traditional steel wires and easier to pass through the bone tunnel; in addition, soft tissue stimulation is limited, and it is easier to pull out when removing the internal fixation.

In this study, compared with the patellotibial tubercle cerclage group, patients of the novel tension band group had a significantly earlier mean partial weight‐bearing time due to advantage (iv) and, thus, an earlier fracture‐healed time; a significantly higher mean Bostman score and knee mobility were achieved at 12‐month follow‐up due to advantage (i) and (iv). Although the operation time of patellotibial tubercle cerclage group was shorter, the position of the patella was more difficult to control in surgical procedure, causing low patella position and high stress in the titanium cable, resulting in a higher incidence of complications.

The necessary precautions during surgery with the new tension band were as follows: (i) broken fracture fragments should remain connected with the soft tissue; (ii) when reducing the fracture, a small clamp should be used to hold the interface between the fragments and the patellar tendon, rather than directly clamping the broken fragments; otherwise, the fragments will be further crushed; (iii) two large fracture fragments of the inferior pole should be selected and fixed with two φ1.5 mm K‐wires inserted from the center of the fracture block as much as possible, 30° from the coronal plane of the patella to avoid the articular surface, not requiring to pass through the proximal end of the patella; (iv) repair of the torn quadriceps dilatation and anterior aponeurosis for fixation strength; (v) the inferior pole patellar fracture does not contain the articular surface. The goal of the operation is to restore the normal knee extension device and achieve bone healing between the fragments, rather than to treat intra‐articular fractures. Postoperative fluoroscopy revealing a small step between the bone blocks does not mean poor functional recovery.

### *Limitations*

This study had some limitations. Firstly, the study design was retrospective and had a small sample size, patients were not randomized into the two treatment groups due to the 5 years period, and this cohort consists of only 63 cases. Secondly, this study only compared the outcomes of the novel tension band to patellotibial tubercle cerclage. No data prove its superiority to other surgical treatments.

### *Conclusions*

Compared with patellotibial tubercle cerclage, patients with comminuted fractures of the inferior pole of the patella treated with a novel tension band experienced longer operation time, but earlier partial weight‐bearing and fracture‐healed time, better clinical outcomes at 12‐month follow‐up, and less complications. Novel tension band should be considered an alternative therapy for the treatment of distal pole patellar fractures.
